# Conserved Structural Motifs at the C-Terminus of Baculovirus Protein IE0 are Important for its Functions in Transactivation and Supporting hr5-mediated DNA Replication

**DOI:** 10.3390/v4050761

**Published:** 2012-05-04

**Authors:** Neta Luria, Liqun Lu, Nor Chejanovsky

**Affiliations:** 1 Entomology Department, Institute of Plant Protection, The Volcani Center, POB 6, Bet Dagan 50250, Israel; Email: neta@volcani.agri.gov.il; 2 Key Laboratory of Exploration and Utilization of Aquatic Genetic Resources (Ministry of Education), College of Fisheries and Life Science, Shanghai Ocean University, Shanghai 201306, China; Email: lqlv@shou.edu.cn

**Keywords:** IE0 transactivator, baculovirus, mutagenesis, functions

## Abstract

IE0 and IE1 are transactivator proteins of the most studied baculovirus, the *Autographa californica* multiple nucleopolyhedrovirus (AcMNPV). IE0 is a 72.6 kDa protein identical to IE1 with the exception of its 54 N-terminal amino acid residues. To gain some insight about important structural motifs of IE0, we expressed the protein and C‑terminal mutants of it under the control of the Drosophila heat shock promoter and studied the transactivation and replication functions of the transiently expressed proteins. IE0 was able to promote replication of a plasmid bearing the *hr5* origin of replication of AcMNPV in transient transfections with a battery of eight plasmids expressing the AcMNPV genes *dnapol*, *helicase*, *lef*-1, *lef-*2, *lef*-3, *p35*, *ie*-2 and *lef*-7. IE0 transactivated expression of the baculovirus *39K* promoter. Both functions of replication and transactivation were lost after introduction of selected mutations at the basic domain II and helix-loop-helix conserved structural motifs in the C-terminus of the protein. These IE0 mutants were unable to translocate to the cell nucleus. Our results point out the important role of some structural conserved motifs to the proper functioning of IE0.

## 1. Introduction

IE0 and IE1, are transactivator proteins of the most studied baculovirus, the *Autographa californica* multiple nucleopolyhedrovirus (AcMNPV). IE1 the widely studied product of the *immediate early gene 1*, is a multifunctional protein of 66.9 kDa involved in regulation of the viral cycle through its ability to transactivate differentially early and late viral genes [1,2,3,4,5,6,7,8,9] and to participate in the replication of the viral genome [10,11]. Thus, IE1 is able to transactivate its own promoter and the promoter of the suppressor of apoptosis *p35* and down regulates the *ie0* promoter [10,12]. 

Transient assays utilizing a combination of a minimal number of plasmids expressing the AcMNPV genes *ie-1*, *dnapol*, *helicase*, *ie-2*, *lef-1*, *lef-2*, *lef-3*, *lef-7* and *p35* indicated that IE1 was involved in AcMNPV DNA replication [10,13].

IE1 displays functionally distinct domains: Two acidic transcriptional activation domains at its N‑terminus, separated by the basic domain I (BDI) which is required for binding to *hr*s, enhancer elements of the AcMNPV genome that function as origins of DNA replication [11,14,15,16] and reviewed in [17]. At the C terminus of IE1 are located the basic domain II (BDII) and a helix-loop-helix domain (HLH). These domains are involved in dimerization, nuclear import and DNA binding [14,18,19,20,21,22]. IE1 interacts with an *hr*-28 mer, the minimal sequence motif required for IE1-mediated enhancer and origin of replication function, as a homodimer [20]. Loss of oligomerization disrupts *hr*‑binding and disturbs IE1 functions [20,21]. Identical *hr* sequences were required for IE1-mediated DNA replication and transactivation of the *hr1a* region of AcMNPV [23]. All the above plus the fact that IE1 localizes to baculovirus DNA replication factories [24] support the idea that IE1 has an important role in baculovirus DNA replication [9].

Much less information is available on the role of IE0, a 74 kDa protein identical to IE1 except for an additional 54 amino acid residues at its N-terminus, thus at least it has the same structural motifs found in IE1. IE0 is the product of the *immediate early gene 0* resulting from splicing of *exon 0* (38 amino acids) to the 5' end of the untranslated leader of the *ie1* mRNA (16 aa and the entire IE-1 582 aa [5,25,26]. Also, IE1 is translated from ie0 *mRNA* from its internal *ie1* start codon. Elimination of the *ie1* start AUG codon by mutating it to GCG prevented translation of IE1 without affecting the function of IE0 [5].

IE0 is expressed early in infection, its level peaks prior to DNA replication and declines late in infection, in contrast to IE1 [5]. 

IE0 is involved in trans-activation of the *ie1* and *polyhedrin* promoters of AcMNPV [5,6], and was shown to upregulate expression of insect and mammalian promoters [27]. Reduced or null expression of *ie0* hampered the ability of AcMNPV to replicate in permissive *S. frugiperda* cells [28,29], but improved AcMNPV replication in non permissive SL2 cells from *S. littoralis* [28,30]. Selective ablation of IE0 by RNAi results in delayed synthesis and lower steady-state levels of IE1 [9] and selective ablation of *ie-1/ie-0* blocked virus DNA synthesis and late gene expression in permissive *Spodoptera frugiperda* cells [9]. Thus, the relative levels of IE0 and IE1 play an important role in a successful AcMNPV infection. 

The *39K* promoter of the baculovirus AcMNPV directs the expression of the *pp31* gene, a prototype “delayed early gene” also referred as *39K* gene, that was expressed at high levels in AcMNPV-infected cells [31,32]. Delayed early genes are dramatically activated by baculovirus early gene products such as IE-1. It has been shown that expression of *pp31/39K* is regulated by tandem early and late promoters [31]. The early promoter consists of dual TATA boxes, a CAGT motif, and upstream regulatory elements [31]. Expression of *39K* in transient assays is dependent on the viral trans-regulatory protein IE1 [32]. Transcription from the late promoter requires the highly conserved TAAG motif [31] and is mediated by the viral-induced RNA polymerase [33]. Transient expression of baculovirus late genes requires the expression of 19 genes referred to as lefs, late expression factors. They include *lef-1* through *lef-12*, *p143*, *p47*, *dnapol*, *p35*, *pp31*, *ie-1*, and *ie-2* [34]. IE1 transactivates the *39K* promoter independently if it is linked in *cis* to an *hr* enhancer motif. In contrast it has been previously shown that IE0 expression transactivates the *39K* promoter in an *hr5* dependent mode only [6]. In the latter study the cloned *ie0* cDNA was expressed from its own promoter. That opens up the possibility that *ie1* was also expressed due to initiation from an internal AUG in the *ie0* mRNA [5]. Moreover, since IE1 was reported to down regulate the expression of *ie0* we decided to directly test the function of IE0 in an IE1-free transient assay in this study (see below). 

From the above, it follows that understanding the function of IE0 may help to elucidate the regulation of AcMNPV infections in permissive and non permissive cells and in this study we took a first step in this direction. It is assumed that, due to their common sequences, IE0 and IE1 share biochemical activities, including *hr* enhancer binding and transcriptional activation [6,7,27]. However, besides the above information very little is known about the functions, structure and cellular localization of IE0. To gain some insight about important structural motifs for the functions of IE0 in an IE1-free environment, we expressed the protein and C-terminal mutants of it under the control of the Drosophila heat shock promoter and studied the transactivation and replication functions of the transiently expressed proteins.

## 2. Results

### 2.1. Functions of IE0

To study the functions of IE0 independently of IE1, we constructed phspie0M, a plasmid that bore *ie0* cDNA under the control of the Drosophila heat shock promoter *hsp70*, and introduced a mutation in the second ATG codon changing it to GCG, thus eliminating translation of IE1 (see Experimental Section). 

**Figure 1:**
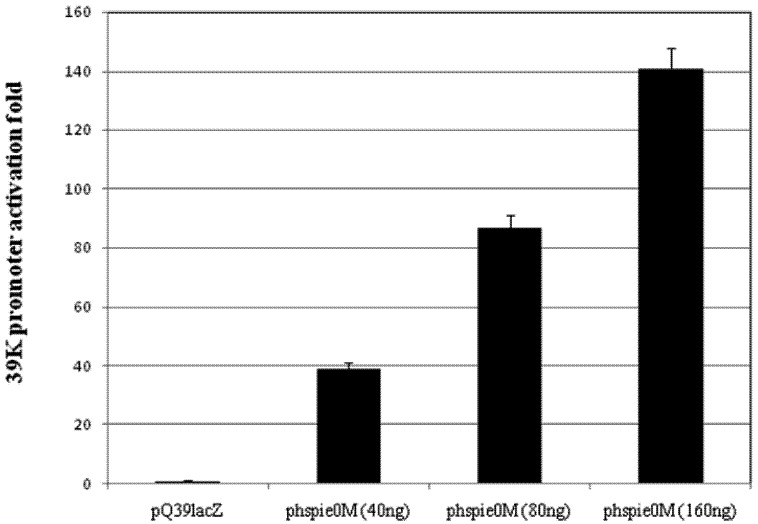
IE0 transactivation of the *39K* promoter. Sf9 1 × 10^5^ cells were transfected with 250 ng pQ39lacZ and increasing amounts of phspie0M (indicated at the bottom). All the transfections were normalized to 750 ng by addition of pBSK. LacZ activity (OD 405 nm) in the cell extracts was determined by standard methods. Results represent duplicate transfections. Bars: standard error.

To determine if IE0, expressed from the above obtained plasmid, phspie0M, was able to transactivate the baculovirus promoter *39K*, we co-transfected variable amounts of phspie0M, namely 40, 80 and 160 ng with a fixed amount of the plasmid p39QlacZ (bearing the *lacZ* gene driven by the *39K* promoter linked in *cis* to the *hr5* enhancer). Expression of *ie0* from the *hsp70* promoter enhanced expression of *lacZ*, by 40, 85 and 140-fold respectively (Figure 1). Co-transfection of 160 ng of phsp70ie1, expressing *ie1* with p39QlacZ resulted in 60 fold activation (not shown).

**Figure 2:**
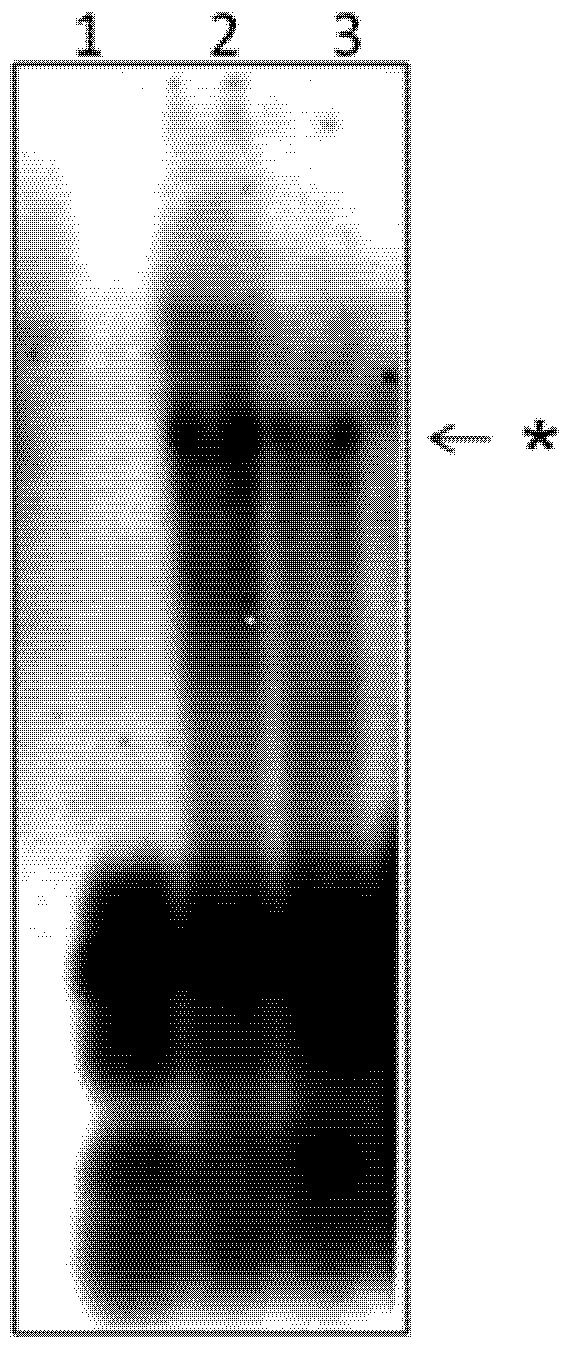
IE0-mediated replication of the *hr5*-containing plasmid pQ35. Sf9 cells were co‑transfected with the replication library containing 500 ng of each plasmid without (lane 1), or with 250 ng of phspie0M (lane 2) or phspie1 (lane 3). Total intracellular DNA was isolated at 72 h posttransfection, digested with DpnI, linearized with XhoI, electrophoresed on a 0.7% agarose gel, and transferred to a GeneScreen Hybridization Transfer Membrane. Hybridization was performed with a Fluorescein dUTP-labeled pQ35 probe. The replicated complexes were detected by chemiluminescence. The samples were digested with XhoI and DpnI. Asterisk, replicated pQ35.

*In vivo* experimentation with mutants of IE0 indicated that it was able to replace to some extent the function of IE1 in AcMNPV replication. To test directly if IE0 was able to replace IE1 in supporting AcMNPV DNA replication, we examined the ability of IE0 to drive replication of the plasmid pQ35 bearing an AcMNPV origin of replication *hr5* in a transient assay. To this end, we replaced phspie1 by phspie0M in Sf9 cells transfected with a library of plasmids bearing the AcMNPV genes required for efficient DNA replication: *dnapol*, *helicase*, *ie-2*, *lef-1*, *lef-2*, *lef-3*, *lef-7*, *p35* (see Experimental section). In the absence of phspie0M the library lacking phspie1 did not replicate the plasmid pQ35 (Figure 2, lane 1). Addition of phspie0M promoted replication of pQ35, as achieved by adding phspie1 (Figure 2, lanes 2 and 3, respectively). 

### 2.2. IE0 Localizes to the Cell Nucleus

**Figure 3:**
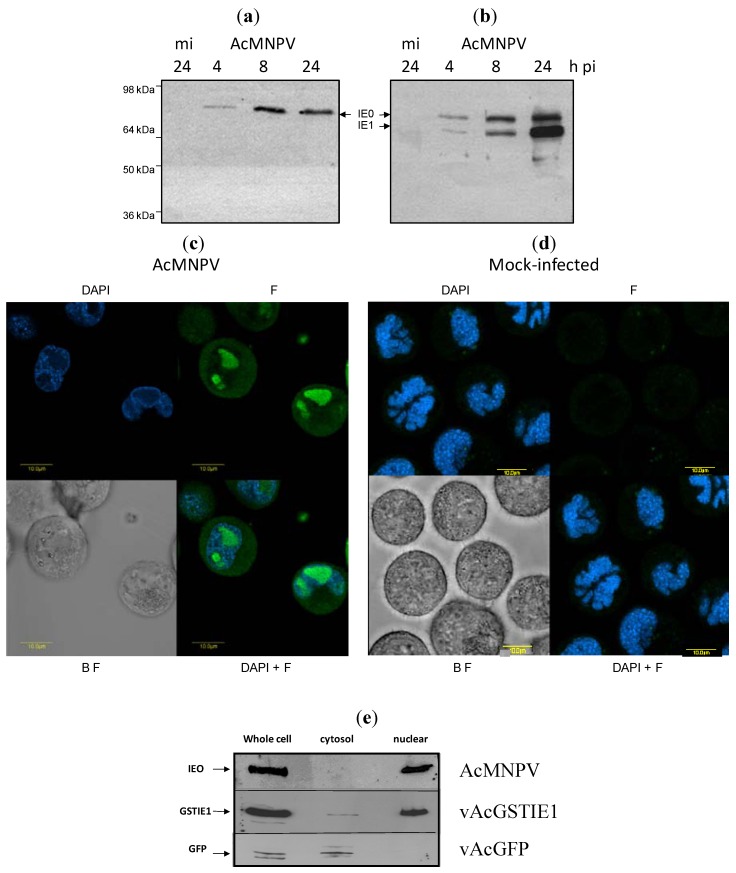
IE0 localizes to the cell nucleus. (**a**) and (**b**) Mock-infected (m.i.) lysed at 24 h- and AcMNPV-infected Sf9 cell extracts collected and lysed at 4, 8 and 24 h post infection. The polypeptides were separated by PAGE and subjected to immunoblot analysis with anti-IE0 (panel a) or anti-IE1/IE0 antiserum (panel b) as described in Experimental section. (**c**) and (**d**) Confocal microscopy analysis of AcMNPV- and mock-infected Sf9 cells, respectively, fixed at 16 h post treatment, incubated with anti-IE0 antiserum and decorated with anti-rabbit IgG fraction Alexa Fluor 488 (F, green); DAPI staining (blue). B F, bright field. Bars, 10 µm. (**e**) Biochemical fractionation of nuclei and cytoplasm from Sf9 cells infected with AcMNPV, vAcGSTIE1 or vAcGFP: cell extracts subjected to Western blot analysis utilizing anti-IE0, anti-GST and anti-GFP antiserum, respectively. Molecular markers are indicated on the left.

Previous reports based on indirect data indicated that IE0 may localize at the nucleus of the cell during infection but no formal demonstration of this ability of IE0 was provided. To answer this question we first produced an anti-IE0 antibody that exclusively recognizes IE0 by expressing its N‑terminal amino acids absent in IE1 (see Experimental Section). Infection of Sf9 cells with AcMNPV allowed us to confirm the specific recognition of IE0 by the anti-IE0 antiserum at various times of infection as compared to the simultaneous recognition of IE0 and IE1 with an anti-IE1/IE0 antiserum previously described, (Figure 3a,b, lanes 4, 8, 24, respectively). Neither of the antisera used reacted with any polypeptide in the mock-infected cells (Figure 3a,b, m.i.) Second, to localize IE0, Sf9 cells were infected with AcMNPV and fixed at 16 h post infection. The fixed cells were immunoreacted with anti-IE0 antiserum and decorated with anti-rabbit IgG fraction Alexa Fluor 488 (green). It can be seen that IE0 localized to the cell nucleus stained by DAPI (blue) in contrast to mock-infected Sf9 cells (Figure 3c,d, respectively). Moreover, Western blot analysis of biochemically fractionated cell nuclei and cytoplasm from a recombinant AcMNPV-infected Sf9 cells confirmed that IE0 localizes at the cell nucleus (Figure 3e, panel AcMNPV). Biochemical fractionation of Sf9 cells infected separately with a recombinant AcMNPV expressing a GST1-tagged IE1 that localizes to the cell nucleus and, with an AcMNPV recombinant expressing GFP that localizes exclusively to the cytoplasm, served as positive and negative controls (Figure 3e, panels labeled vAcGSTIE1 and vAcGFP, respectively). 

### 2.3. C-Terminal Mutants of IE0

**Figure 4:**
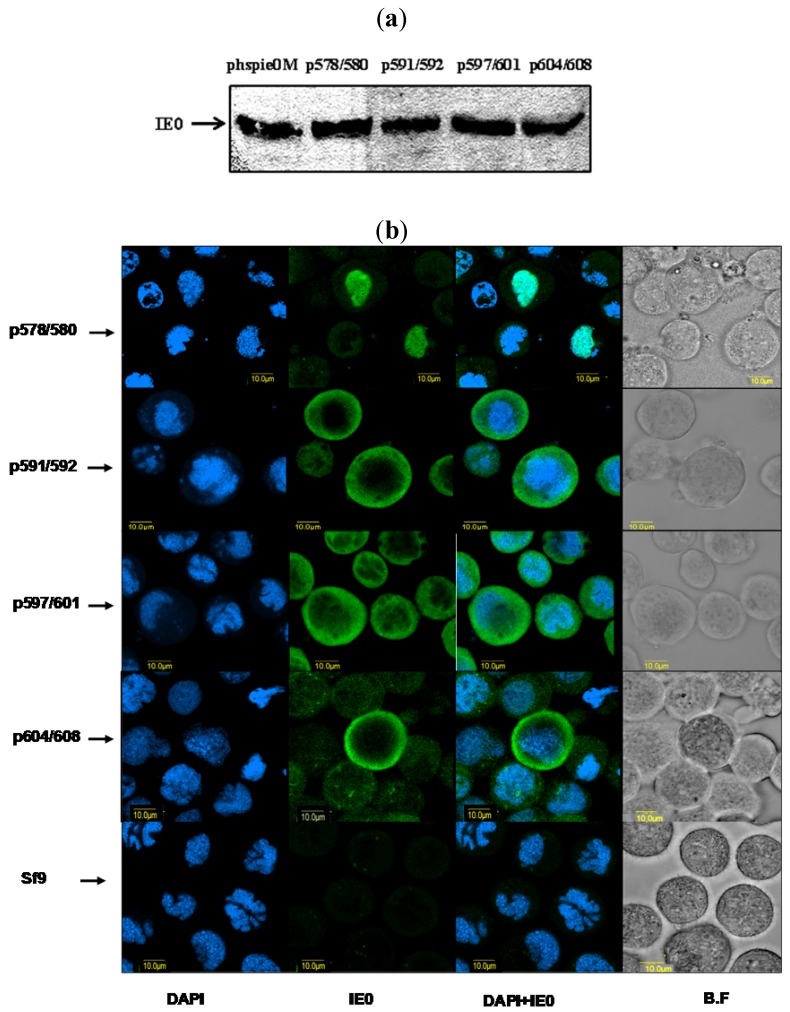
Cellular localization of C-terminal mutants of IE0. (**a**) Western blot analysis of Sf9 cells transfected with the plasmid constructs phspIE0M, p578/580, p591/592, p597/601 or p604/608 (indicated above the figure). (**b**) Confocal microscopy analysis. Sf9 cells were transfected with the plasmids p578/580, p591/592, p597/601 or p604/608 (arrows) or mock-transfected (Sf9) and treated as described in Figure 3. The fluorescence and bright light channels are indicated at the bottom. Bars, 10 µm.

Since IE0 bears a Basic domain (BDII) and a helix-loop-helix domain (HLH), C-terminal motifs present in IE1, we introduced selected mutations into these motifs and studied their effect on the functions of IE0. Thus, we constructed four different double mutants of IE0: two mutants in the BDII motif, R^578^A/K^580^A (p578/580) and R^591^A/R^592^A (p591/592); and two mutants in the HLH motif, L^597^D/L^601^E (p597/601) and L^604^D/I^608^E (p604/608). Expression of the mutant IE0 proteins was confirmed by immunoblot in Sf9 cells transiently transfected with the corresponding plasmids (Figure 4a). Then, we studied the ability of the mutants of IE0 to enter the cell nucleus following transfection of Sf9 cells with their corresponding plasmids. As can be seen, only mutant IE0 expressed from p578/580 was able to enter the cell nucleus in contrast to the mutants expressed from p591/592, p597/601 and p604/608 (Figure 4b).

**Figure 5:**
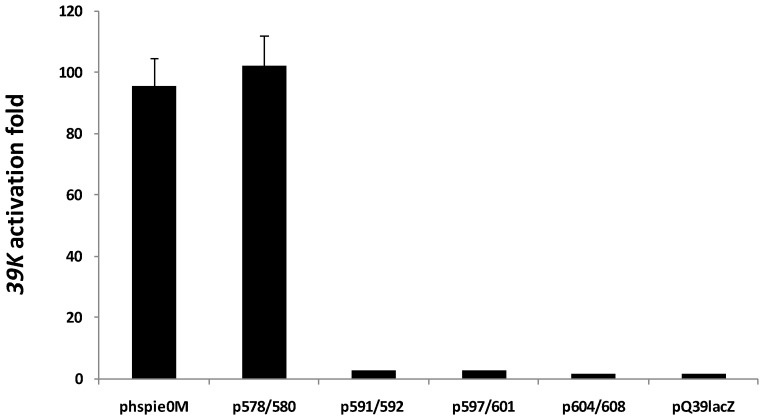
Transactivation of the *39K* promoter by C-terminal mutants of IE0. Fold of transactivation of lacZ expression from cells cotransfected with 250 ng pQ39lacZ and 80 ng of each the constructs indicated at the bottom. The samples were processed as indicated in Figure 1. Results represent duplicate transfections. Bars: standard error.

**Figure 6:**
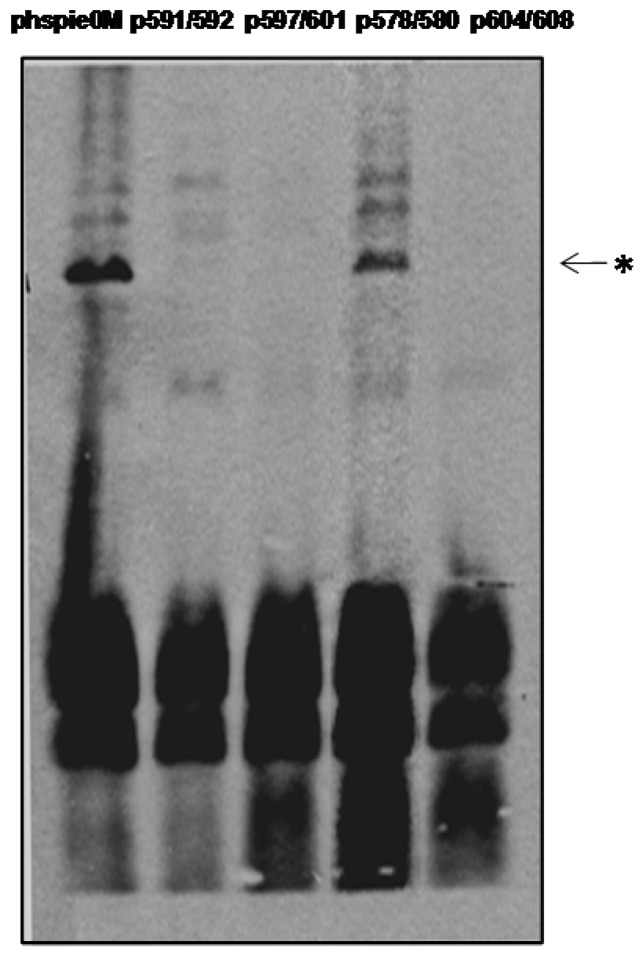
The ability of the various C-terminal mutants of IE0 to mediate replication of the hr5-containing plasmid pQ35. Sf9 cells were co-transfected with the replication library plasmids as described in Figure 1 (500 ng of each plasmid) with 250 ng of phspie0M or each of the mutants indicated above the figure. Total intracellular DNA was isolated at 72 h posttransfection, digested with DpnI, linearized with XhoI, electrophoresed on a 0.7% agarose gel, and transferred to a GeneScreen Hybridization Transfer Membrane. Hybridization was performed with a Fluorescein dUTP-labelled pQ35 probe. The replicated complexes were detected by chemiluminescence. The samples were digested with XhoI and DpnI. Asterisk, replicated pQ35.

To evaluate whether the mutant proteins were able to perform the functions of IE0, we examined their ability to transactivate the *39K* promoter and to promote replication of the *hr5*-bearing plasmid in our transient-transfection assays described above. Only p578/580 was able to transactivate expression from the *39K* promoter (Figure 5). Moreover, this mutant was the only one able to replicate the plasmid pQ35 in our transient replication assay (Figure 6). A summary of the properties of the IE0 mutants is shown in Table 1.

**Table 1:** Properties of the IE0 C-terminal mutants expressed from their corresponding transfected plasmids.

IE0-Mutant	Cellular localization	Transactivation of *39K* promoter	Replication of *hr5*-plasmid
R^578^A/K^580^A (p578/580)	Nucleus	+	+
R^591^A/R^592^A (p591/592)	Cytoplasm	−	−
L^597^D/L^601^E (p597/601)	Cytoplasm	−	−
L^604^D/I^608^E (p604/608)	Cytoplasm	−	−

**Figure 7:**
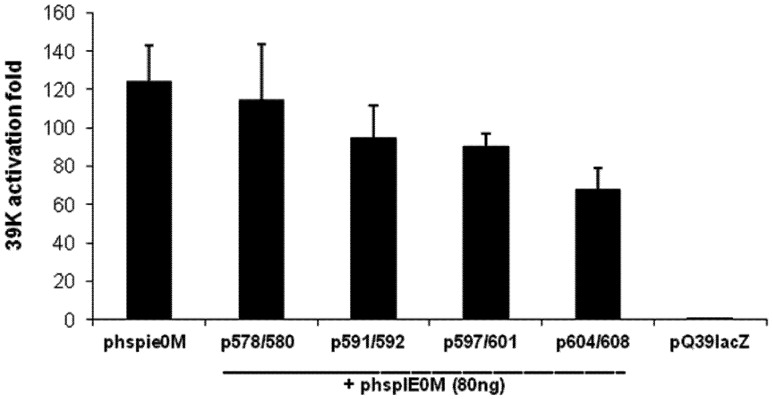
Effect of co-transfection of C-terminal mutants of IE0 and IE0 on lacZ expression from the *39K* promoter. Sf9 cells were transfected with pQ39lacZ (250 ng) and phspie0M alone (160 ng), or with phspie0M (80 ng) and each one of the constructs indicated below the figure (80 ng). All the transfections were treated as described above. Results represent duplicate transfections. Error bars represent standard errors.

Finally, we studied the function of these mutants of IE0 when they were co-transfected with wild type IE0 in the p39QlacZ transactivation assay. Co-transfection of the plasmid mutant p578/580 with phspie0M (80 ng of each plasmid) activated p39QlacZ to the same extent of activation achieved by transfecting a total equal amount of phspie0M (160 ng). On the other hand, replacing the latter mutant with either p591/592; p597/601 or p604/608 resulted in 76 to 51% inhibition of the transactivation ability of IE0 (Figure 7). 

## 3. Experimental Section

### 3.1. Cell Lines and Viruses

*S. frugiperda* Sf9 cells were maintained and propagated in TNM-FH medium supplemented with 10% heat-inactivated fetal bovine serum and infected with wild-type AcMNPV E-2 strain as described previously [35,36].

vAcGSTIE1, is a recombinant virus bearing an extra copy of *ie1* engineered in the polyhedrin locus of AcMNPV using pFastBac as a transfer vector. This extra copy of *ie1* expresses the GST epitope fused in frame to the N-terminus of IE1. vAcGFP was described before [37].

### 3.2. Plasmids and Site Directed Mutagenesis

Phspie1 and phspie0 bearing the *ie1* and *ie0* coding regions under the control of the *D. melanogaster hsp70* promoter were described before [28,38]. phspie0M was obtained by replacing the second ATG of the *ie0* open reading frame of phspie0 by GCG (Met to Ala) utilizing oligonucleotide-site directed mutagenesis with the primers IE1M3-CCAAGTGACTGCGACGCAAATTA and IE1M3R-TTGCGTCGCAGTCACTTGGTTG and the methodology described previously [28,39]. Changing the above Met to Ala in IE0 was shown to abolish synthesis of IE1 and we confirmed that result ([5] and not shown). Similarly, C-terminus mutants of IE0 were constructed utilizing the primers: 578/F580CCGATTACAAACATATGATGCGCAAATGCATTATATTTTTG and 578/580R CAAAAATATAATGCATTTGCGCATCATATGTTTGTAATCGG; 591/592F GCAATGTAGTGCTCTCTGCTGCGTTAACTTTACCGATTAC; and 591/592R GTAATCGGTAAAGTTAACGCAGCAGAGAGCACTACATTGC; 597/601F TAAAGCTAACAATTTGAGCTCATTATTGTGGTCTGTAGTGCTCTC; and 597/601R GAGAGCACTACAGACCACAATAATGAGCTCAAATTGTTAGCTTTA; 604/608F CGGAACCAGACCCTGGAGCTCTAAAGCTAAGTCTTTTAACAAATTATTG and 604/608R CAATAATTTGTTAAAAGACTTAGCTTTAGAGCTCCAGGGTCTGGTTCCG.

pET-IE0^1–44^, an expression plasmid of *exon0* (encoding IE0^1–44^), was constructed using the T7 expression vector pET22b (−) (Novagen). Since the *ie0* ORF contains 2 exons (*exon 0* and *exon1*), a hybrid-PCR strategy was used for the fusion of *exon0* and *exon1* as described before [28]. The exon*0*‑exon*1* fused fragment obtained was digested with *Eco*RI and *Hinc*II, ligated to the *Eco*RI, *Sma*I site of pBSK vector to obtain pBSKexon0. Then the *Eco*RI-*Not*I fragment from pBSKexon0 was cloned to the corresponding sites in pET 22b (−) vector to obtain pET-IE0^1–44^. In pET-IE0^1–44^, *exon0* was in frame with the His_6_-tag ORF at the C-terminus and the *pe1B* leader sequence at the N-terminus and expression was controlled by the T7 promoter, thus it expresses exclusively the IE0 N-terminal amino acids absent in IE1. 

pQ35, that contains the complete *p35* gene with its own promoter and a *hr5* enhancer, was derived by inserting the corresponding *Xho*I-*Sac*I fragment of about 3 kb from the AcMNPV genome to pBSK.

pQ39LacZ, in which the *LacZ* gene was under the control of viral *39K* promoter linked with a *hr5* enhancer: the *LacZ* gene was cut from pCMVβ (Clontech) with BamH I and inserted to the BamH I site of vector p39QCAT [1] replacing the *cat* ORF.

### 3.3. Preparation of Anti-IE0 Specific Antiserum

Expression in *E. coli*, transformed with pET-IE0^1–44^, was induced by adding IPTG and incubating the culture overnight at 37 °C. A bacterial lysate was obtained and the His-tagged recombinant peptide purified by affinity chromatography on Ni^2+^ beads as described before [35]. Protein purity was more than 90% as judged by SDS-PAGE (not shown). New Zealand white rabbits were immunized and antiserum was prepared commercially using standard procedures (Hylabs, Il). 

### 3.4. Transient Transfection Assays

Transient β-galactosidase (LacZ) assay. 1–2 × 10^5^ Sf9 cells were transfected with the reporter plasmid pQ39LacZ and the tested plasmids (phspie1, phspie0 or phspie0M). LacZ reporter assays were described before [40]. The experiments were performed in duplicates, three biological repetitions.

Transient DNA replication assays were performed following the methodology previously published [10] and by utilizing pQ35, that served not only as a plasmid expressing *p35* but also as a reporter plasmid in the replication assay since it contained the replication origin *hr5* (see below). The replication library consisted of nine pBSK-based vectors bearing the AcMNPV genes *dnapol*, *helicase*, *lef*-1, *lef-*2, *lef*-3, *p35*, *ie*-2 and *lef*-7, *ie1*, and p35 required for efficient transient DNA replication [2,5,10,41,42]. *p35* was expressed from pQ35. *Dpn*I digestion was used to differentiate between input plasmid from bacterial containing methylated deoxyadenosine residues and plasmid DNA replicated in the insect cells [10]. Sf9 (2.6 × 10^6^) cells were co-transfected with the replication library containing 500 ng of each plasmid (with or without out phspie1), and various amounts of phspie0M. At 4 h post transfection, the cells were heat shocked at 42 °C for 30 minutes. Total intracellular DNA was isolated at 72 h post transfection, digested with *Dpn*I, linearized with *Xho*I, electrophoresed on a 0.7% agarose gel, and transferred to a GeneScreen Hybridization Transfer Membrane (NEN^TM^ Life Science products, Inc., Boston, MA, USA)). Hybridization was performed with a Fluorescein dUTP‑labelled pQ35 probe. Probe-labeling, hybridization and signal detection were performed according to the protocol of the Renaissance random primer Fluorescein labeling kit (NEN^TM^). The replicated complexes were detected by chemiluminescence. 

### 3.5. Western Blot Analysis

Virus-infected (MOI of 5) or plasmid-transfected cells were harvested and subjected to SDS‑polyacrylamide gel electrophoresis (PAGE) with a pH 9 separating gel; this procedure allowed us to distinguish clearly the IE0 molecular species from the IE1 molecular species. Immunoblot analysis was performed with anti-IE0/IE1 [30], anti-IE0 (1:1000 dilution), anti-GFP and anti-GST, as previously described [30].

### 3.6. Confocal Microscopy

Sf9 cells (2 × 10^5^) were infected with AcMNPV, or transfected with plasmids encoding IE0 or IE0 mutants. The cells were collected at 16 h postinfection or at 48 h post-transfection, washed with PBS, and fixed with 2% formaldehyde, 0.2% glutaraldehyde for 5 min at 4 °C. Subsequently, the cells were washed with PBS and permeabilized with 0.15% Triton X-100 in PBS for 10 min, washed in PBS, blocked with 2% BSA in PBS for 1 h at RT and finally incubated with anti-IE0 or mouse anti-GST antisera in blocking solution. The fixed cells were subsequently incubated with anti rabbit IgG fraction Alexa Fluor488 (Invitrogen) for 30 min. Nuclear staining was performed with DAPI. The cells were subsequently washed in PBS and observed by confocal microscopy. Microscopy was performed with an Olympus IX 81 inverted laser scanning confocal microscope. Three dishes per transfection were used and the experiments were repeated twice.

### 3.7. Biochemical Fractionation

Sf9 cells (4 × 10^5^) were infected with AcMNPV, vAcGSTIE1 or vAcGFP, or transfected with plasmids encoding IE0 or the above IE0 mutants or IE1. The cells were collected at 16 h postinfection or at 48 h post-transfection, washed with PBS, and lysed by suspension in in 170 µL of TBN buffer (10 mM Tris [pH 6.5], 140 mM NaCl, 0.5% NP-40, 3 mM MgCl_2_, protease inhibitors) for 15 min on ice. After clarification by centrifugation at 5,220 *g*, 5 min, the supernatant was retained as the cytosolic fraction. The pellet was washed with TBN buffer (250 µL), lysed by suspension for 15 min on ice with 50 µL of lysis buffer (62.5 mM Tris [pH 6.8], 40% glycerol, 4% sodium dodecyl sulfate [SDS], 3% dithiothreitol), and forced 10 to 20 times through a 25-gauge needle. After clarification by centrifugation (16,000 *g*, 10 min), the supernatant was retained as the nuclear fraction. To analyze total cell proteins, a sample of the cell suspension was removed prior to cell lysis and mixed with an equal volume of 2% β-mercaptoethanol-2% SDS. All the fractions obtained were subjected to SDS‑PAGE and immunoblot analysis. The experiment was repeated three times (duplicate transfections per sample).

## 4. Discussion and Conclusions

The role of IE0 in the AcMNPV infectious cycle is not very clear. Former studies revealed that IE0 steady state levels were higher early in infection and lower at later times, in contrast to the levels of IE1 [28,29,30]. Reduced expression of *ie0* compared to *ie1* enhanced the ability of AcMNPV to replicate in non-permissive *S. littoralis* SL2 cells [30]. A recombinant AcMNPV expressing i*e0* but not *ie1* replicated less efficiently in permissive *S. frugiperda* Sf9 cells [29]. Also, previous work suggested that IE0 may bind to viral DNA and that it may interact with IE1 modulating the viral infection [7,28,29,30]. Thus, we decided to study the functional properties of IE0 as an initial step to characterize it in the absence of IE1 and to facilitate later investigation of its interaction with IE1. 

We examined for the first time directly the ability of IE0 to transactivate the baculovirus *39K* promoter and promote DNA replication independently of the presence of IE1 by using transient assays. We found that under our experimental conditions the *39K* early promoter was transactivated about 85 fold by IE0. That degree of activation was stronger than what we obtained with similar amounts of IE1 under these conditions (not shown). Although *39K* is an early and late promoter, the transient assay we used monitored the ability of IE0 to transactivate the early function of *39K*, since its late function requires the presence of another 18 late expression factors [34]. 

We show that IE0 enters the cell nucleus and is able to sustain replication of a plasmid bearing a baculovirus origin of replication, *hr5* (Figure 1, Figure 2, Figure 3). These data provide direct support about the ability of IE0 to mediate replication of AcMNPV DNA that was inferred from experiments done with mutants defective or null in IE1 using *in vivo* studies [28,29,30]. 

IE0 diplays at its C-terminus a basic motif (BDII, amino acids 575 to 592) rich in arginine and lysine and a helix-loop-helix motif (HLH, amino acids 597–622). To assess the contribution of these two structural motifs to the functionality of IE0 we introduced:

1. Two charged to neutral mutations at the residues 578 and 580 (mutant p578/580), and 591–592 (mutant p591/592) of the BDII motif and,
2. Two hydrophobic to charged mutations at residues 597 and 601 (p597/601) and 604 and 608 (p604/608) of the HLH motif.

Three of the mutants, p591/592, p597/601 and p604/608, were unable to enter the cell nucleus, to promote transactivation of the *39K* promoter and to induce DNA replication (Figure 4, Figure 5, Figure 6 and Table 1) showing that the BDII amino acid residues proximal to the HLH motif and those belonging to the latter motif are essential to maintain a functional IE0. The C-terminus of IE0 and IE1 are identical and both proteins share the same motifs. The basic BDII and HLH domains of IE1 were shown to be involved in dimerization, nuclear import and DNA binding [19,20,21]. Disruption of these HLH domains in IE1 resulted in loss of oligomerization in contrast to disruption of the residues R537 and R538 present in its BDII domain that resulted in a protein that retained the oligomerization ability but that was unable to enter the cell nucleus [20]. Concomitantly, these mutants lost the functions of IE1-mediated DNA binding to a 28-mer fragment of *hr5* and transactivation of the *p35* promoter driven by the *hr5* region of AcMNPV [20,21]. The functional properties of our parallel mutants p591/592, p597/601 and p604/608 described above are similar to those of their corresponding IE1 mutants. Thus, though in this study we did not directly address the ability of IE0 to form dimers one possible explanation for the reduced activity of transactivation observed by mixing the functional inactive mutant p591/592 with phspie0M expressing wild type IE0, is that the former may inhibit entrance to the nuclei of hetero-dimeric IE0 (mutant-wild type) by forming dimers that present only a partial nuclear localization signal to the cell or viral proteins involved in nuclear transport [21]. 

IE1 binding to *hr5* required the presence of another intact basic domain I motif (amino acid residues 152 to 161 [14]. Loss of this *hr5* binding property of IE1 resulted in its failure to promote transactivation of the *p35* promoter [14]. The same motif is present in IE0 and was not modified in our HLH-mutants p597/601 and p604/608. Thus we can hypothesize that these mutants may have retained their putative ability to bind to *hr5* but as described above they were not functional for transactivation, DNA replication and nuclear entrance. If that is the case, we should have expected that these mutants would compete with wild type IE0 in *hr5*-binding when co-transfected with it causing a reduction in the degree of transactivation of the *39K* promoter, and this is what we have observed (Figure 7). An alternative explanation is that the degree of transactivation observed corresponds just to the net input of wild type IE0 in the experiment and there is no additional interaction between these mutants and wild type proteins implicating that the N-terminal domain of IE0 may block the dimerization properties imprinted in the BDI domain it shares with IE1. Further studies addressing the involvement of the N-terminal domain of IE0 in *hr5* binding are required to validate these hypotheses.

In summary, this study provides information about the functioning of IE0 in transactivation and DNA replication in the absence of IE1, as well as the contribution of structural elements of its C‑terminus to these functions, and as such it helps to characterize better IE0 for a better understanding of its role in the cell infected with AcMNPV.
